# Supplementary material for: Effects of replacement therapies with clotting factors in patients with hemophilia: A systematic review and meta-analysis
Source: PLoS One. 2022 Jan 14;17(1):e0262273. doi: 10.1371/journal.pone.0262273 (PMC8759703; doi:10.1371/journal.pone.0262273)
Supplement: S1 Table — (PDF) [file pone.0262273.s001.pdf]

Supporting information

S1 Table. Search strategy.

Search engine	Date	Term	Results
PubMed	December 10, 2020	(haemophilia[TIAB] OR hemophilia[TIAB] OR "Hemophilia A"[Mesh] OR "Hemophilia B"[Mesh] OR "Factor XI Deficiency"[TIAB] OR "Factor VIII Deficiency"[TIAB]) AND (prophyla*[TIAB] OR episodic[TIAB] OR target[TIAB]) AND ((clinical[TIAB] AND trial[TIAB]) OR "clinical trials as topic"[MeSH] OR "clinical trial"[PT] OR random*[TIAB] OR “random allocation”[MeSH])	502
Scopus	December 11, 2020	(TITLE-ABS ("haemophilia") OR TITLE-ABS ("hemophilia") OR INDEXTERMS ("Hemophilia A") OR INDEXTERMS ("Hemophilia B") OR TITLE-ABS ("Factor XI Deficiency") OR TITLE-ABS ("Factor VIII Deficiency")) AND (TITLE-ABS("prophyla*") OR TITLE-ABS ("episodic") OR TITLE-ABS ("target")) AND ((TITLE-ABS ("clinical") AND TITLE-ABS ("trial")) OR INDEXTERMS ("clinical trials as topic") OR DOCTYPE ("clinical trial") OR TITLE-ABS	521

		("random*") OR INDEXTERMS ("random allocation"))	
CENTRAL	December 12, 2020	#1 (haemophilia):ti,ab,kw<br>#2 (hemophilia):ti,ab,kw<br>#3 #1 OR #2<br>#4 (prophyla*):ti,ab,kw<br>#5 (episodic):ti,ab,kw<br>#6 (target*):ti,ab,kw<br>#7 #4 OR #5 OR #6<br>#8 #3 AND #7	540
