# Supplementary material for: Effects of replacement therapies with clotting factors in patients with hemophilia: A systematic review and meta-analysis
Source: PLoS One. 2022 Jan 14;17(1):e0262273. doi: 10.1371/journal.pone.0262273 (PMC8759703; doi:10.1371/journal.pone.0262273)
Supplement: S2 Table — (PDF) [file pone.0262273.s002.pdf]

Supporting information

S2 Table: Studies that were evaluated in full-text, and were excluded.

N°	Title	Exclusion reason
1	Antunes S, Tangada S, Stasyshyn O, Mamonov V, Phillips J, Guzman-Becerra N, et al. A prospective, open-label, randomized, parallel study with AICC to evaluate the efficacy and safety of prophylactic vs. on-demand treatment in hemophilia A or B subjects with inhibitors. Journal of thrombosis and haemostasis: JTH. 2013.	Were not studies published in scientific journals (conference abstracts, protocols, letter to the editor, or narrative reviews)
2	Antunes SV, Tangada S, Phillips J, Stasyshyn O, Mamonov V, Guzman-Becerra N, et al. Comparison of historic on-demand versus prospective on-demand and prophylaxis bleeding episodes in hemophilia A and B patients with inhibitors treated with FEIBA NF. Haemophilia. 2014.	Were not studies published in scientific journals (conference abstracts, protocols, letter to the editor, or narrative reviews)
3	Björkman S. Pharmacokinetics of plasma-derived and recombinant factor IX - implications for prophylaxis and on-demand therapy. Haemophilia. 2013.	Not a randomized control trial.
4	Carcao M, Feldman B, Pazmino-Canizares J, Hilliard P, Stain A, Blanchette V. Results of a randomized prospective clinical trial comparing standard to	Were not studies published in scientific journals (conference abstracts, protocols, letter to the editor, or narrative reviews)

	tailored prophylaxis in severe hemophilia A. Haemophilia. 2006.	
5	Chakraborty J, Bhattacharyya M, Sankar Ray S. Effect of low dose prophylaxis versus episodic treatment on joint events in severe hemophilia a patients above 2 years: a randomized open label pilot study in Eastern India. Indian journal of hematology & blood transfusion. 2018.	Were not studies published in scientific journals (conference abstracts, protocols, letter to the editor, or narrative reviews)
6	Chozie N, Primacakti F, Tulaar A, Setiabudy R, Prasetyo M, Gatot D. Low-dose prophylaxis versus on-demand treatment in Indonesian children with severe hemophilia A: an interim report. Haemophilia. 2018.	Were not studies published in scientific journals (conference abstracts, protocols, letter to the editor, or narrative reviews)
7	Chuansumrit A, Isarangkura P, Hathirat P. Prophylactic treatment for hemophilia A patients: a pilot study. The Southeast Asian journal of tropical medicine and public health. 1995.	Were not studies published in scientific journals (conference abstracts, protocols, letter to the editor, or narrative reviews)
8	Chuansumrit A, Sosothikul D, Natesirinilkul R, Lektrakul Y, Charoonruangrit U. Efficacy and safety of low-dose prophylaxis of highly purified plasma-derived factor VIII concentrate produced by the National Blood Centre, Thai Red Cross Society. Haemophilia. 2018.	Not a randomized control trial.
9	Collins P, Faradji A, Morfini M, Enriquez MM, Schwartz L. Efficacy and safety of secondary	Not a randomized control trial.

	prophylactic vs. on-demand sucrose-formulated recombinant factor VIII treatment in adults with severe hemophilia A: results from a 13-month crossover study. Journal of thrombosis and haemostasis: JTH. 2010.	
10	Ducore JM, Lalezari S, Santagostino E, Ahuja SP, Enriquez MM, Tueckmantel C, et al. Improvement in quality of life outcomes and bleeding rates among patients switching from on-demand FVIII to prophylactic bay 94-9027 in the protect VIII study. Blood. 2019.	Were not studies published in scientific journals (conference abstracts, protocols, letter to the editor, or narrative reviews)
11	Elalfy M, Elbarbary N. Bleeding patterns in severe hemophilia A infants and toddlers on prophylaxis vs. on-demand therapy: a prospective randomized observational study. Haemophilia. 2012.	Were not studies published in scientific journals (conference abstracts, protocols, letter to the editor, or narrative reviews)
12	EU/EEA EO. Study to compare on-demand treatment with 2 prophylaxis regimens of BeneFIX in severe Hemophilia B subjects. 2015.	Were not studies published in scientific journals (conference abstracts, protocols, letter to the editor, or narrative reviews)
13	Euctr BG. Trial to evaluate the effect of two treatment regimens for adults with severe hemophilia A. 2009.	Were not studies published in scientific journals (conference abstracts, protocols, letter to the editor, or narrative reviews)
14	Euctr CZ. A phase II/III, randomized, cross-over, open-label trial to demonstrate superiority of	Were not studies published in scientific journals (conference

	prophylaxis over on-demand therapy in previously treated subjects with severe hemophilia A treated with plasma protein-free recombinant FVIII formulated with sucrose (BAY 81-8973). 2010.	abstracts, protocols, letter to the editor, or narrative reviews)
15	Euctr CZ. Randomized, comparative, open label treatment with double-blind placebo-controlled periods within treatment study to evaluate the efficacy and safety of a once-a-week prophylaxis treatment with BAY 79-4980 compared to once-a-week prophylaxis treatment and to on-demand treatment with rFVIII-FS reconstituted with water for injection in previously treated patients with severe hemophilia A. 2006.	Were not studies published in scientific journals (conference abstracts, protocols, letter to the editor, or narrative reviews)
16	Euctr DE. A Multicenter, Open-Label Study to Compare On-Demand Treatment With 2 Prophylaxis Regimens of Recombinant Coagulation Factor IX (BeneFIX) Reformulated Drug Product (rFIX-R) in Subjects with Severe Hemophilia B. - Prophylaxis Clinical Study. 2007.	Were not studies published in scientific journals (conference abstracts, protocols, letter to the editor, or narrative reviews)
17	Euctr ES. A Phase III Study to Evaluate the Efficacy, Safety, and Pharmacokinetics of Prophylactic RO5534262 versus No Prophylaxis in Hemophilia A Patients with Inhibitors. 2015.	Were not studies published in scientific journals (conference abstracts, protocols, letter to the editor, or narrative reviews)
18	Euctr FR. A Double-Blind, Randomized, Crossover Study of the Recovery of FACTANE 100 versus 200	Were not studies published in scientific journals (conference

	IU/mL followed by an Open-Label Safety Study of FACTANE 200 IU/mL in Previously Treated Patients With Severe (FVIII: C<1%) Haemophilia A. 2009.	abstracts, protocols, letter to the editor, or narrative reviews)
19	Euctr IT. A diagnostic interventional, controlled, cross-sectional evaluation of joint status using MRI in subjects with severe hemophilia A treated with primary prophylaxis, secondary prophylaxis, or on-demand therapy - ND. 2009.	Were not studies published in scientific journals (conference abstracts, protocols, letter to the editor, or narrative reviews)
20	Euctr IT. Advate Antihemophilic Factor (Recombinant), Plasma/Albumin-Free Method (Advate rAHF?PFM): a Phase 4 Study Comparing Two Prophylactic Regimens In Subjects With Severe Or Moderately Severe Hemophilia A. - ADVATE: a Phase IV Prophylactic Study. 2009.	Were not studies published in scientific journals (conference abstracts, protocols, letter to the editor, or narrative reviews)
21	Euctr IT. An Investigator-sponsored study on rFVIIa prophylaxis in children with haemophilia A and inhibitors - ENJOIH 01. 2009.	Were not studies published in scientific journals (conference abstracts, protocols, letter to the editor, or narrative reviews)
22	Euctr IT. Feiba nf: studio prospettico in aperto, randomizzato, a gruppi paralleli, volto a valutare efficacia e sicurezza del trattamento profilattico vs quello al bisogno in pazienti affetti da emofilia a o b e con inibitori a titolo elevato - FEIBA NF Prophylaxis Study. 2010.	Were not studies published in scientific journals (conference abstracts, protocols, letter to the editor, or narrative reviews)

23	Euctr IT. Phase I/II/III Pharmacokinetic and outcome study of Inspiration`s Recombinant Factor IX product, IB1001, in subjects with Hemophilia B - ND. 2009.	Were not studies published in scientific journals (conference abstracts, protocols, letter to the editor, or narrative reviews)
24	Euctr NL. A Multi-centre, Single-blind Trial Evaluating Safety and Efficacy, including Pharmacokinetics, of NNC-0156-0000-0009 when used for Treatment and Prophylaxis of Bleeding Episodes in Patients with Haemophilia B - paradigm™2. 2011.	Were not studies published in scientific journals (conference abstracts, protocols, letter to the editor, or narrative reviews)
25	Euctr NL. Randomized, active-controlled, double-blind, parallel design study to evaluate the efficacy and safety of a once-a-week prophylaxis treatment with BAY 79-4980 compared to three times-per-week prophylaxis with rFVIII-FS in previously treated patients with severe hemophilia A. 2007.	Were not studies published in scientific journals (conference abstracts, protocols, letter to the editor, or narrative reviews)
26	Euctr PL. A study of the Safety and Effectiveness of Pegylated Recombinant Factor VIII (BAX 855) in Prevention of Bleeding in Patients with Severe Hemophilia A (a blood clotting disorder) using two different dosing schedules to target different levels of BAX855 in the blood. 2016.	Were not studies published in scientific journals (conference abstracts, protocols, letter to the editor, or narrative reviews)
27	Gringeri A, Lundin B, Von Mackensen S, Mantovani L, Mannucci P. A randomized clinical trial on prophylaxis Vs. episodic treatment in children with	Were not studies published in scientific journals (conference

	haemophilia A: the ESPRIT study. Haemophilia. 2010.	abstracts, protocols, letter to the editor, or narrative reviews)
28	Gringeri A, Lundin B, von Mackensen S, Mantovani LG, Mannucci PM, Bianchi Bonomi A. Primary and secondary prophylaxis in children with haemophilia A reduces bleeding frequency and arthropathy development compared to on-demand treatment: a 10-year, randomized, clinical trial. Proceedings of the XXII congress of the international society on thrombosis and haemostasis (ISTH), boston, MA, USA, 11-16th july 2009. 2009.	Were not studies published in scientific journals (conference abstracts, protocols, letter to the editor, or narrative reviews)
29	Holme PA, Poulsen L, Simpson M, Liu T, Maas Enriquez M, Pabinger-Fasching I. Overall annualised bleeding rates <2 in patients with haemophilia A treated with every-5-day or every-7-day BAY 94-9027 prophylaxis for ≥100 exposure days. Haemophilia. 2019.	Were not studies published in scientific journals (conference abstracts, protocols, letter to the editor, or narrative reviews)
30	Jo H, Gringeri A, Leissinger C, Mantovani L, Cortesi P, Gemmen E. Evaluating the cross-over effect on health-related quality of life in a randomized cross-over study of hemophilia-a patients. Value in health. 2011.	Were not studies published in scientific journals (conference abstracts, protocols, letter to the editor, or narrative reviews)
31	Kavakli K, Smith L, Kuliczkowski K, Korth-Bradley J, You CW, Fuiman J, et al. Once-weekly prophylactic treatment vs. on-demand treatment with	Not a randomized control trial.

	nonacog alfa in patients with moderately severe to severe haemophilia B. Haemophilia. 2016.	
32	Kavakli K, Yang R, Rusen L, Beckmann H, Tseneklidou-Stoeter D, Enriquez MM. Superiority of prophylaxis versus on-demand therapy with plasma protein-free recombinant FVIII formulated with sucrose (BAY 81-8973): LEOPOLD II study results. Haemophilia. 2014.	Were not studies published in scientific journals (conference abstracts, protocols, letter to the editor, or narrative reviews)
33	Kempton C, Valluri S, Reding M, Lissitchkov T, Goranov S, Gercheva L, et al. Randomized, controlled, parallel-group trial of secondary prophylaxis vs on-demand treatment with sucrose-formulated recombinant factor VIII in adults with severe hemophilia A. Haemophilia. 2012.	Were not studies published in scientific journals (conference abstracts, protocols, letter to the editor, or narrative reviews)
34	Kempton C, Valluri S, Reding M, Lissitchkov T, Goranov S, Gercheva L, et al. Regional differences in baseline patient-reported outcomes in a randomized, controlled, prospective trial of secondary prophylaxis VS on-demand treatment in patients with severe hemophilia A. Haemophilia. 2012.	Were not studies published in scientific journals (conference abstracts, protocols, letter to the editor, or narrative reviews)
35	Kerlin B, Kenet G, Saxena K, Caicedo J, Wang M, Afonja O. Baseline characteristics and bleeding outcomes in patients with severe hemophilia a administered bay 94-9027 prophylaxis every 5 or every 7 days. Pediatric blood & cancer. 2017.	Were not studies published in scientific journals (conference abstracts, protocols, letter to the editor, or narrative reviews)

36	Klamroth R, St Ledger K, Blackman N, Veldman A, Santagostino E, Oldenburg J, et al. Dosing of rVIII-singlechain based on clinical bleeding phenotypes: low bleeding rates and low consumption in patients treated with prophylaxis two or three times weekly. Haemophilia Conference: 10th annual congress of the european association for haemophilia and allied disorders France Conference start: 20170201 Conference end: 20170203. 2017.	Were not studies published in scientific journals (conference abstracts, protocols, letter to the editor, or narrative reviews)
37	Konkle B, Friedrich U, Abrams Z. Secondary prophylactic treatment with rFVIIa in patients with haemophilia A or B and inhibitors with high requirements for on-demand treatment. Haemophilia. 2006.	Were not studies published in scientific journals (conference abstracts, protocols, letter to the editor, or narrative reviews)
38	Konkle BA, Stasyshyn O, Chowdary P, Bevan DH, Mant T, Shima M, et al. Pegylated, full-length, recombinant factor VIII for prophylactic and on-demand treatment of severe hemophilia A. Blood. 2015.	Not a randomized control trial.
39	Kulkarni R, Karim FA, Glamocanin S, Janic D, Vdovin V, Ozelo M, et al. Results from a large multinational clinical trial (guardian™3) using prophylactic treatment with turoctocog alfa in paediatric patients with severe haemophilia A: Safety, efficacy and pharmacokinetics. Haemophilia. 2013.	Not a randomized control trial.

40	Kulkarni R, Ragni MV, Baker RI, Pasi J, Manco-Johnson MJ, Potts J. Analysis of clinical target joint bleeding on prophylactic regimens with recombinant Factor VIII Fc fusion protein in patients with severe hemophilia A. Haemophilia. 2014.	Were not studies published in scientific journals (conference abstracts, protocols, letter to the editor, or narrative reviews)
41	Lindvall K, Astermark J, Björkman S, Ljung R, Carlsson KS, Persson S, et al. Daily dosing prophylaxis for haemophilia: a randomized crossover pilot study evaluating feasibility and efficacy. Haemophilia. 2012	Not a randomized control trial.
42	Ljung R, Kenet G, Mancuso ME, Kaleva V, Rusen L, Tseneklidou-Stoeter D, Michaels LA, Shah A, Hong W, Maas Enriquez M; investigators of the LEOPOLD Kids Trial. BAY 81-8973 safety and efficacy for prophylaxis and treatment of bleeds in previously treated children with severe haemophilia A: results of the LEOPOLD Kids Trial. Haemophilia. 2016	Not a randomized control trial.
43	Mahlangu JN, Kavakli K, Hvitfeldt Poulsen L, Tseneklidou-Stoeter D, Beckmann H, Enriquez MM, et al. Prophylactic efficacy of twice-weekly vs. 3-timesweekly bay 81-8973 in severe hemophilia A: results of the Leopold I and II clinical trials. Journal of thrombosis and haemostasis: JTH. 2015.	Were not studies published in scientific journals (conference abstracts, protocols, letter to the editor, or narrative reviews)
44	Nct. A Trial to Compare Prophylaxis Therapy to On-demand Therapy With a New Full Length	Were not studies published in scientific journals (conference

	Recombinant FVIII in Patients With Severe Hemophilia A. 2010.	abstracts, protocols, letter to the editor, or narrative reviews)
45	Nct. Efficacy and Safety Study of Prophylactic Versus On-Demand Treatment With Feiba NF in Subjects With Hemophilia A or B and a High Titer Inhibitor. 2009.	Were not studies published in scientific journals (conference abstracts, protocols, letter to the editor, or narrative reviews)
46	Nct. Efficacy of NovoSeven® in Bleeding Prophylaxis in Hemophilia. 2005.	Were not studies published in scientific journals (conference abstracts, protocols, letter to the editor, or narrative reviews)
47	Nct. Efficacy Study of Activated Prothrombin Complex for Prevention of Bleeds in Hemophilia A With Inhibitors. 2005.	Were not studies published in scientific journals (conference abstracts, protocols, letter to the editor, or narrative reviews)
48	Nct. Prophylaxis Study of Recombinant Factor VIII Manufactured Protein-Free (rAHF-PFM) in Patients With Hemophilia A. 2005.	Were not studies published in scientific journals (conference abstracts, protocols, letter to the editor, or narrative reviews)
49	Nct. Safety and Efficacy of 3 Different Doses of Long Acting Factor VII in Haemophilia A or B Patients With Inhibitors. 2009.	Were not studies published in scientific journals (conference abstracts, protocols, letter to the editor, or narrative reviews)
50	Nct. Study Comparing On-Demand Treatment With Two Prophylaxis Regimens Of BeneFIX In Patients With Severe Hemophilia B. 2006.	Were not studies published in scientific journals (conference

		abstracts, protocols, letter to the editor, or narrative reviews)
51	Nct. Study of the Efficacy of Human Recombinant Factor VIII (Kogenate FS) Reconstituted in Pegylated Liposomes. 2005.	Were not studies published in scientific journals (conference abstracts, protocols, letter to the editor, or narrative reviews)
52	Nct. The Effect of Patient Counseling on Adolescent Hemophilia Patient Compliance With Bleeding Logs. 2005.	Were not studies published in scientific journals (conference abstracts, protocols, letter to the editor, or narrative reviews)
53	Nct. Trial to Evaluate the Effect of Secondary Prophylaxis With rFVIII Therapy in Severe Hemophilia A Adult and/or Adolescent Subjects Compared to That of Episodic Treatment. 2008.	Were not studies published in scientific journals (conference abstracts, protocols, letter to the editor, or narrative reviews)
54	Nugent D, O'Mahony B, Dolan G. Value of prophylaxis vs on-demand treatment: application of a value framework in hemophilia. Haemophilia. 2018.	Not a randomized control trial.
55	Oldenburg J, Holme P, Ducore J, Maas Enriquez M, Wang M. Annualised bleeding rates ≤2 with every-5-day and every-7-day dosing of BAY 94-9027 in the protect VIII study. Haemophilia. 2019.	Were not studies published in scientific journals (conference abstracts, protocols, letter to the editor, or narrative reviews)
56	Pocoski J, Humphries TJ, Hong W. Spinart 3-year data: patient-reported benefits of adult prophylaxis vs on-demand treatment with bayer's sucrose-	Were not studies published in scientific journals (conference abstracts, protocols, letter to the editor, or narrative reviews)

	formulated recombinant factor viii. Haemophilia. 2015.	
57	Pollmann W, Huppertz-Helmhold S. Comparison of prophylaxis and on-demand treatment of hemophilia A patients treated with refacto. Annals of hematology. 2001.	Were not studies published in scientific journals (conference abstracts, protocols, letter to the editor, or narrative reviews)
58	Powell JS, Perry D, Baker RI, Shapiro A, Manco-Johnson M, Potts J. Analysis of clinical target joint bleeding on prophylactic regimens with recombinant Factor IX Fc fusion protein in patients with severe hemophilia B. Haemophilia. 2014.	Were not studies published in scientific journals (conference abstracts, protocols, letter to the editor, or narrative reviews)
59	Ragni M, Kessler C, Fogarty P, Josephson N, Neff A, Raffini L. Phase III randomized, controlled trial of pre-emptive vs. standard prophylaxis to prevent inhibitor formation in children with severe hemophilia A: the inhibit study. Haemophilia. 2011.	Were not studies published in scientific journals (conference abstracts, protocols, letter to the editor, or narrative reviews)
60	Ragni MV, Yabes JG, Fogarty PF, Josephson NC, Kessler CM, Neff AT, et al. Pilot randomized, non-inferiority, cross-over trial of once-weekly vs. three times-weekly recombinant factor VIII prophylaxis in adults with severe haemophilia A. Haemophilia. 2017.	Were not studies published in scientific journals (conference abstracts, protocols, letter to the editor, or narrative reviews)
61	Ragni MV. Rationale for a randomized controlled trial comparing two prophylaxis regimens in adults	Not a randomized control trial.

	with severe hemophilia A: the Hemophilia Adult Prophylaxis Trial. Expert Rev Hematol. 2011	
62	Rendo P, Smith L, Korth-Bradley J, Charnigo R, Shafer F. Evaluation of two secondary prophylaxis regimens of recombinant factor IX (r-fix) in moderately severe to severe (fix <2%) hemophilia B patients. Haemophilia. 2013.	Were not studies published in scientific journals (conference abstracts, protocols, letter to the editor, or narrative reviews)
63	Rendo P, Smith L, Lee HY, Shafer F. Nonacog alfa: An analysis of safety data from six prospective clinical studies in different patient populations with haemophilia B treated with different therapeutic modalities. Blood Coagulation Fibrinolysis. 2015.	Not a randomized control trial.
64	Riva S, Gringeri A, Fusco F, Mantovani L. Direct costs of children with haemophilia A undergoing prophylaxis or episodic treatment: results from the ESPRIT study. Haemophilia. 2012.	Were not studies published in scientific journals (conference abstracts, protocols, letter to the editor, or narrative reviews)
65	Santagostino E, Gringeri A, Muça-Perja M, Mannucci PM. A prospective clinical trial of implantable central venous access in children with haemophilia. Br J Haematol. 1998.	Not a randomized control trial.
66	Shapiro AD, Neufeld EJ, Blanchette V, Salaj P, Gut RZ, Cooper DL. Safety of recombinant activated factor VII (rFVIIa) in patients with congenital haemophilia with inhibitors: Overall rFVIIa exposure and intervals following high (>240 μg kg-1)	Not a randomized control trial.

	rFVIIa doses across clinical trials and registries. Haemophilia. 2014.	
67	Smith Mark P, Rupon J, Wali Y, Remawi H, Korth-Bradley J, Smith L, et al. Impact of Treatment Regimen with Moroctocog Alfa (AF-CC) on Bleeding Frequency in Pediatric Aged Subjects with Moderately Severe to Severe Hemophilia A. Blood. 2018.	Were not studies published in scientific journals (conference abstracts, protocols, letter to the editor, or narrative reviews)
68	Song CK, Quon DV, Dolan G, Recht M, Guzman-Becerra N, Epstein JD, et al. Access to prophylaxis benefits hemophilia A subjects in both low and high IU/capita countries: adult subset analysis of a multicenter, open-label, randomized, phase 4 clinical study of rAHF-PFM. Haemophilia. 2012.	Were not studies published in scientific journals (conference abstracts, protocols, letter to the editor, or narrative reviews)
69	Songkla PN, Seksarn P, Sosothikul D. Comparison of different prophylactic regimens for Thai children with moderate and severe haemophilia A. Haemofilia. 2020.	Not a randomized control trial.
70	Tiede A, Oldenburg J, Lissitchkov T, Knaub S, Bichler J, Manco-Johnson MJ. Prophylaxis vs. on-demand treatment with Nuwiq(®) (Human-cl rhFVIII) in adults with severe haemophilia A. Haemophilia. 2016.	Not a randomized control trial.
71	Urasinski T, Stasyshyn O, Andreeva T, Rusen L, Perina FG, Oh MS, et al. Recombinant factor IX	Not a randomized control trial.

	(BAX3261) in previously treated paediatric patients with haemophilia B: A prospective clinical trial. Haemophilia. 2015.	
72	Valentino LA, Ewenstein BM, Guzman-Becerra N, Wong WY, Schoenig-Diesing C, Romanov V. Bleeding pattern and median time interval between bleeding episodes amongst patients receiving on-demand and prophylaxis therapy. Journal of thrombosis and haemostasis: JTH. 2013.	Were not studies published in scientific journals (conference abstracts, protocols, letter to the editor, or narrative reviews)
73	Valentino LA, Plushch O, Rusen L, Andreeva T, Elezovic I, Charnigo R. A multicenter, open-label study to compare on-demand treatment with two prophylaxis regimens of recombinant coagulation factor IX in subjects with hemophilia B. Journal of thrombosis and haemostasis: JTH. 2011.	Were not studies published in scientific journals (conference abstracts, protocols, letter to the editor, or narrative reviews)
74	Verma S, Dutta T, Mahadevan S, Nalini P, Biswal N, Basu D, et al. Low-dose Factor VIII prophylaxis in children with severe hemophilia. Haemophilia. 2014.	Were not studies published in scientific journals (conference abstracts, protocols, letter to the editor, or narrative reviews)
75	Von MacKensen S, Riva S, Fusco F, Mantovani L, Gringeri A. Quality of life of children with hemophilia A undergoing prophylaxis or episodic treatment: results from the ESPRIT study. Journal of thrombosis and haemostasis: JTH. 2011.	Were not studies published in scientific journals (conference abstracts, protocols, letter to the editor, or narrative reviews)

76	Wu R, Sun J, Xiao J, Liu Y, Xue F, Wang H, et al. A prospective study of health-related quality of life of boys with severe haemophilia A in China: comparing on-demand to prophylaxis treatment. Haemophilia. 2017.	Not a randomized control trial.
77	Zarkova AG, Kempton CL, Reding MT, Lissitchkov T, Goranov S, Gercheva L. Bleeding frequency subanalyses in a randomized controlled trial of prophylaxis vs on-demand treatment with sucrose-formulated recombinant factor VII in adults with severe hemophilia A. Haemophilia. 2013.	Were not studies published in scientific journals (conference abstracts, protocols, letter to the editor, or narrative reviews)
78	Zhao Y, Xiao J, Yang R, Wu R, Hu Y, Beckmann H, et al. Efficacy of standard prophylaxis versus on-demand treatment with bayer's sucrose-formulated recombinant FVIII (rFVIII-FS) in Chinese children with severe hemophilia A. Pediatric hematology and oncology. 2017.	Not a randomized control trial.
79	Corrigendum to Randomized, controlled, parallel-group trial of routine prophylaxis vs. on-demand treatment with sucrose-formulated recombinant factor VIII in adults with severe hemophilia A (SPINART). Journal of thrombosis and haemostasis : JTH. 2014.	Not a randomized control trial.
80	Santagostino E, Mancuso ME, Rocino A, Mancuso G, Scaraggi F, Mannucci PM. A prospective randomized	Did not assess the interventions of interest

	trial of high and standard dosages of recombinant factor VIIa for treatment of hemarthroses in hemophiliacs with inhibitors. J Thromb Haemost. 2006.	
81	Antunes SV, Tangada S, Stasyshyn O, Mamonov V, Phillips J, Guzman-Becerra N, et al. Randomized comparison of prophylaxis and on-demand regimens with FEIBA NF in the treatment of haemophilia A and B with inhibitors. HAEMOPHILIA. 2014.	Did not assess the interventions of interest
82	Ljung, R., Karim, F. A., Saxena, K., Suzuki, T., Arkhammar, P., … Rosholm, A. 40K glycoPEGylated, recombinant FVIIa: 3-month, double-blind, randomized trial of safety, pharmacokinetics and preliminary efficacy in hemophilia patients with inhibitors. Journal of Thrombosis and Haemostasis. 2013.	Did not assess the interventions of interest
