# Supplementary material for: Effects of replacement therapies with clotting factors in patients with hemophilia: A systematic review and meta-analysis
Source: PLoS One. 2022 Jan 14;17(1):e0262273. doi: 10.1371/journal.pone.0262273 (PMC8759703; doi:10.1371/journal.pone.0262273)
Supplement: S3 Table — *The risk in the intervention group (and its 95% confidence interval) is based on the assumed risk in the comparison group and the relative effect of the intervention (and its 95% CI). CI: Confidence interval; eMRI: Extended magnetic resonance imaging; MD: mean difference; IQR: Interquartile range; SD: Standard deviation; HRQoL: Health-related quality of life. Explanations a. We rated down one level for risk of bias. b. We rated down two levels for imprecision due to the small number of participants that presented the outcome (less than 200). c. We rated down one level for publication bias. (PDF) [file pone.0262273.s003.pdf]

Supporting information

S3 Table. Additional results found in the studies.

**Outcomes (follow-up)**	**№ of participants (studies)**	**Anticipated absolute effects* (95% CI)**		**Relative effect (95% CI)**	**Certainty of the evidence (GRADE)**
		**Risk with Control**	**Risk with Intervention**		
Episodic treatment vs Low-dose prophylaxis					
Change in the Haemophilia Early Arthropathy Detection with Ultrasound (HEAD-US) (12 months)	50 (1 RCT)	• **Chozie 2019:** Low-dose prophylaxis: mean of 1.84. Episodic treatment: mean of 0.2 (p= 0.257).			⊕◯◯◯ VERY LOW [a,b,c]
Episodic treatment vs Intermediate-dose prophylaxis					
Change in the Colorado Adult Joint Assessment Scale (CAJAS), which assess joint physical	83 (1 RCT)	• **Manco-Johnson 2017:** Intermediate-dose prophylaxis: mean –0.31. Episodic treatment: mean 0.63 (p= 0.007).			⊕◯◯◯ VERY LOW [a,b,c]

examination (lower = better) (36 months)			
Pain score (Short-form McGill Pain Questionnaire) (lower = better) (36 months)	83 (1 RCT)	• **Manco-Johnson 2017:** Intermediate-dose prophylaxis: mean –2.5. Episodic treatment: mean 2.4. No p-value provided.	⊕◯◯◯ VERY LOW [a,b,c]
Change in activity level (36 months)	83 (1 RCT)	• **Manco-Johnson 2017** reported that the proportions at baseline and at 3 years were:<br>○ Increased activity: Intermediate-dose prophylaxis 7.1% to 40.5%. Episodic treatment: 14.3% to 19.0%.<br>○ Unrestricted work/school and recreational activities: Intermediate-dose prophylaxis: 33.3% to 47.6%. Episodic treatment: 38.1% to 26.2%.	⊕◯◯◯ VERY LOW [a,b,c]
Healthcare Resource Utilization (HRU) (36 months)	83 (1 RCT)	• **Manco-Johnson 2017:** HRU in patients with episodic treatment was higher than in those with intermediate-dose prophylaxis: hematologists (2.43-fold), primary care physicians (3.17-fold), and nurses (2.2-	⊕◯◯◯ VERY LOW [a,b,c]

		fold); laboratory utilization (1.79-fold); and joint surgeries (performed in 23.8% vs 9.5% of patients).	
Treatment satisfaction (36 months)	83 (1 RCT)	• **Manco-Johnson 2017** reported that the treatment satisfaction at 3 years in patients with intermediate-dose prophylaxis and episodic treatment were:<br>o Treatment somewhat/greatly exceeded their expectations: 42.9% and 26.2%.<br>o Very/extremely satisfied with treatment: 64.3% and 42.9%.<br>o Probably/definitely would continue with their replacement therapies: 66.7% and 47.6%.	⊕◯◯◯ VERY LOW [a,b,c]
Studies that compared two different prophylactic factors			
Annualized bleeding rate (ABR) (11.5 months)	131 (1 RCT)	• **Powell 2012:** ABR was higher (worse) in patients with BAY 79-4980 (mean 15.0 SD 14.2) than in those with rFVIII-FS (mean 5.8 SD 6.9). No p-value provided.	⊕◯◯◯ VERY LOW [a,b,c]
Annualized joint bleeding rate (AJBR) (11.5 months)	131 (1 RCT)	• **Powell 2012:** AJBR was higher (worse) in patients with BAY 79-4980 (mean 12.2 SD 14.2) than in those with rFVIII-FS (mean 5.0 SD 6.3). No p-value provided.	⊕◯◯◯ VERY LOW [a,b,c]

Adverse events (11.5 months)	131 (1 RCT)	• **Powell 2012:** No patient developed inhibitors in both groups.	⊕◯◯◯ VERY LOW [a,b,c]
Studies that assessed intermediate-dose prophylaxis vs pharmacokinetic prophylaxis			
Annualized bleeding rate (ABR) (12 months)	66 (1 RCT)	• **Valentino 2012:** Mean ABR in patients with PK-prophylaxis: 1.9. Intermediate-dose prophylaxis: 1.6, p= 0.2588.	⊕◯◯◯ VERY LOW [a,b,c]
Quality of life (12 months)	66 (1 RCT)	• **Valentino 2012:** median score of HRQoL at 1 year in patients in both groups treatment “did not show statistical differences” (no data provided).	⊕◯◯◯ VERY LOW [a,b,c]
Adverse events (12 months)	66 (1 RCT)	• **Valentino 2012** reported that no patient developed inhibitors in both groups, and that the adverse events was similar in patients with PK-prophylaxis (mean rate 0.089) and in those with intermediate-dose prophylaxis (mean rate 0.356), p> 0.05.	⊕◯◯◯ VERY LOW [a,b,c]
Studies in which the groups received the same weekly doses but with different frequency (50 IU/kg twice a week, vs 100 IU/kg once a week)			
Annualized bleeding rate (ABR) (8 months)	44 (1 RCT)	• **Valentino 2014:** Mean ABR was similar in patients that used 50 IU/kg twice a week: 2.6. In patients that used 100 IU/kg once a week: 4.6, p = 0.217.	⊕◯◯◯ VERY LOW [a,b,c]

Annualized joint bleeding rate (8 months)	44 (1 RCT)	• **Valentino 2014** Mean AJBR in patients that used 50 IU/kg twice a week: 1.9. In patients that used 100 IU/kg once a week: 3.6. No p-value provided.	⊕◯◯◯ VERY LOW [a,b,c]

*The risk in the intervention group (and its 95% confidence interval) is based on the assumed risk in the comparison group and the relative effect of the intervention (and its 95% CI).

CI: Confidence interval; **eMRI**: Extended magnetic resonance imaging; **MD**: mean difference; **IQR**: Interquartile range; **SD**: Standard deviation; **HRQoL**: Health-related quality of life.

Explanations

a. We rated down one level for risk of bias.
b. We rated down two levels for imprecision due to the small number of participants that presented the outcome (less than 200).
c. We rated down one level for publication bias.
