# Supplementary material for: Effects of replacement therapies with clotting factors in patients with hemophilia: A systematic review and meta-analysis
Source: PLoS One. 2022 Jan 14;17(1):e0262273. doi: 10.1371/journal.pone.0262273 (PMC8759703; doi:10.1371/journal.pone.0262273)
Supplement: Attachment — Submitted filename: Response to Reviewers.docx [file pone.0262273.s005.docx]

**Dear editor**

We have carefully considered the views and have reviewed the application accordingly. Our responses are presented below each of the reviewer's comments.

**Journal requirements:**

- **1. Please ensure that your manuscript meets PLOS ONE's style requirements, including those for file naming. The PLOS ONE style templates can be found at https://journals.plos.org/plosone/s/file?id=wjVg/PLOSOne_formatting_sample_main_body.pdf and https://journals.plos.org/plosone/s/file?id=ba62/PLOSOne_formatting_sample_title_authors_affiliations.pdf.**
  - We agree, so we change the manuscript to PLOS ONE’s style:
    - We changed the heading format, and used level 1 heading in bold type, 18pt font in all major sections.
    - We changed the heading format, and used level 2 heading in bold type, 16pt font in all sub-sections of major sections.
    - We changed the heading format, and used level 3 heading in bold type, 14pt font in all sub-sections within Level 2 headings.
    - We changed double-space the entire document and enumerated each line as the example format.
    - We changed the figure citations to Fig 1, Fig 2, etc. Figure captions now appear directly after the paragraph in which they were first cited. We saved a file in tiff format, named Fig1.tiff, Fig2.tiff, and Fig3.tiff.
    - We added the table citations and tables directly after the paragraph in which they were first cited.
    - We changed the format supporting information citations as S1 Table, S2 Table, S3 Table. Then, we listed supporting information captions at the end of the manuscript. Finally, we saved the files as S1_Table.pdf.
    - We created other document with the title, authors, and affiliations according to the suggested format.
  - We put all the changes in red.
- **2. Please include your tables as part of your main manuscript and remove the individual files. Please note that supplementary tables (should remain/ be uploaded) as separate "supporting information" files**
  - We agree, so we added the table citations and tables directly after the paragraph in which they were first cited. Then we deleted the individual files. On the other hand, the supporting information was kept in a different file, and they were listed at the end of the manuscript.
- **3. We note that this manuscript is a systematic review or meta-analysis; our author guidelines therefore require that you use PRISMA guidance to help improve reporting quality of this type of study. Please upload copies of the completed PRISMA checklist as Supporting Information with a file name “PRISMA checklist”.**
  - We agree, so we rename the document to “PRISMA checklist.” In addition, the number described in each item was updated, due to the changes that we were made in the document.
- **4. Thank you for submitting the above manuscript to PLOS ONE. During our internal evaluation of the manuscript, we found significant text overlap between your submission and the following previously published works, some of which you are an author (https://journals.plos.org/plosone/article?id=10.1371%2Fjournal.pone.0233220)
  We would like to make you aware that copying extracts from previous publications, especially outside the methods section, word-for-word is unacceptable. In addition, the reproduction of text from published reports has implications for the copyright that may apply to the publications.
  Please revise the manuscript to rephrase the duplicated text, cite your sources, and provide details as to how the current manuscript advances on previous work. Please note that further consideration is dependent on the submission of a manuscript that addresses these concerns about the overlap in text with published work.
  We will carefully review your manuscript upon resubmission, so please ensure that your revision is thorough.**
  - We agree, so we entered the entire manuscript in *quetext.com*, to verify matches in the sentences. Then, we rephrase some sentences to reduced coincidences. However, some phrases describe the meaning of PRISMA, GRADE, or domains of the risk of bias assessment, or subsection that each systematic review, that we cannot modify. Please tell us if there is more we can do to improve this topic.
- **Please review your reference list to ensure that it is complete and correct. If you have cited papers that have been retracted, please include the rationale for doing so in the manuscript text, or remove these references and replace them with relevant current references. Any changes to the reference list should be mentioned in the rebuttal letter that accompanies your revised manuscript. If you need to cite a retracted article, indicate the article’s retracted status in the References list and also include a citation and full reference for the retraction notice.**
  - We agree, so we checked all the references in the manuscript and there were no retractions. Then we verified the correct reference citation of each one.

**Reviewer #1:**

- **R1C1: This is an interesting and well-written paper. The conclusion is not unexpected as the benefits of prophylaxis are well established this paper has the merit to provide an update on current status of knowledge.**
  - We thank you for acknowledging the relevance of this work.
- **R1C2: Maybe the authors should discuss and compare relative data on SHL and EHL-FVIII.**
  - We agree, so we have included the following paragraph in the discussion (right before the “strengths and limitations” subheading:
    - “All studies found in our systematic review assessed interventions that used CFCs with standard half-life. CFCs with extended half-life require fewer CFCs administration per week due to their pharmacokinetic properties, which may improve the quality of life of the patients; and also require less use of medical devices, which could help lower the cost of prophylactic treatment. Thus, there is a need of RCTs assessing the use of extended half-life CFCs, which are believed to be as safe and effective as standard half-life CFCs, as WFH 2020 suggests. [6]”
- **R1C3: In the title + abstract clearly indicate that only HA is covered.**
  - We understand that our main results are based on hemophilia A, but this search aimed to find studies in **patients with either hemophilia A or B**. Although only one study on hemophilia B was found, and this has been described in the table 1 (characteristics of the studies).
- **R1C4: A table with a summary of what information should be collected in future trials could be useful.**
  - We agree that this information is valuable, so we have included the following paragraph in the discussion (right before the “conclusion” subheading):
    - “These limitations reflect the need for high-quality RCTs that compare different doses of prophylactic treatment (low, intermediate, or high doses) or extended half-life vs standard half-life CFCs. Which assess clinically relevant outcomes (mortality, ABR, ABJR, joint disease, joint status, pain, current health status-HRQol, activities, employment, educational attendance, resource utilization) [43], and with enough follow-up period to assess these outcomes (ideally more than one year).”

**Reviewer #2:**

- **R2C1:** **This is an elegant review and meta-analysis by Taype-Rondan on the effect of clotting factor substitution in patients with haemophilia. The criteria of meta-analyses were met.**
  - We thank you for your kind comment.
- **R2C2:** **It would still be interesting to see the authors' outlook on a possible future study design of trials that include the effect of long-acting factor preparation and nonfactor therapy.**
  - We agree that this information is valuable, so we have included the following paragraph in the discussion (right before the “conclusion” subheading):
    - “These limitations reflect the need for high-quality RCTs that compare different doses of prophylactic treatment (low, intermediate, or high doses) or extended half-life vs standard half-life CFCs. Which assess clinically relevant outcomes (mortality, ABR, ABJR, joint disease, joint status, pain, current health status-HRQol, activities, employment, educational attendance, resource utilization) [43], and with enough follow-up period to assess these outcomes (ideally more than one year).”
